# Age- and muscle-specific reliability of muscle architecture measurements assessed by two-dimensional panoramic ultrasound

**DOI:** 10.1186/s12938-021-00967-4

**Published:** 2022-02-13

**Authors:** Iris Hagoort, Tibor Hortobágyi, Nicolas Vuillerme, Claudine J. C. Lamoth, Alessio Murgia

**Affiliations:** 1 Present Address: Department of Human Movement Sciences, University Medical Center Groningen, University of Groningen, Groningen, The Netherlands; 2 Université Grenoble Alpes, AGEIS, Grenoble, France; 3 Institute of Sport Sciences and Physical Education, Faculty of Sciences, University of Pécs, Pecs, Hungary; 4 Somogy County Kaposi Mór Teaching Hospital, Kaposvár, Hungary; 5 Division of Training and Movement Sciences, Research Focus Cognition Sciences, University of Potsdam, Potsdam, Germany; 6 Institut Universitaire de France, Paris, France; 7 LabCom Telecom4Health, Orange Labs & Univ. Grenoble Alpes, CNRS, Inria, Grenoble INP-UGA, Grenoble, France

**Keywords:** Ultrasonography, Aging, Echo intensity, Sarcopenia, Reliability, Muscle architecture

## Abstract

**Background:**

Age-related changes in muscle properties affect daily functioning, therefore a reliable assessment of such properties is required. We examined the effects of age on reliability, muscle quality and interrelation among muscle architecture (MA) parameters of the gastrocnemius medialis (GM), tibialis anterior (TA), and vastus lateralis (VL) muscles.

**Methods:**

Three raters scored ultrasound (US) scans of 12 healthy younger and older adults, on fascicle length (FL), pennation angle (PA) and muscle thickness (MT). Intra- and inter-rater reliability of MA measures in rest and contraction was assessed by intraclass correlation coefficients (ICC) and standard error of measurements (SEM, SEM%). The relationship between MA parameters was examined using Pearson correlation coefficients. Muscle quality (MQ) was examined using mean pixel intensity.

**Results:**

Reliability was moderate to excellent for TA in both groups (ICCs: 0.64–0.99, SEM% = 1.6–14.8%), and for VL in the younger group (ICCs: 0.67–0.98, SEM% = 2.0–18.3%). VL reliability was poor to excellent in older adults (ICCs: 0.22–0.99, SEM% = 2.7–36.0%). For GM, ICCs were good to excellent (ICCs: 0.76–0.99) in both groups, but GM SEM% were higher in older adults (SEM%_Younger_ = 1.5–10.7%, SEM%_Older_ = 1.6–28.1%). Muscle quality was on average 19.0% lower in older vs. younger adults. In both groups, moderate to strong correlations were found for VL FL and MT (*r* ≥ 0.54), and TA PA and MT (*r* ≥ 0.72), while TA FL correlated with MT (*r* ≥ 0.67) in younger adults only.

**Conclusions:**

In conclusion, age- and muscle-specificities were present in the relationships between MT and PA, and MT and FL at rest. Furthermore, the reliability of MA parameters assessed with 2D panoramic US is acceptable. However, the level of reliability varies with age, muscle and MA measure. In older adults notably, the lowest reliability was observed in the VL muscle. Among the MA parameters, MT appears to be the simplest and most easily reproducible parameter in all muscles and age groups.

**Supplementary Information:**

The online version contains supplementary material available at 10.1186/s12938-021-00967-4.

## Background

Healthy aging is associated with a transformation of muscle composition [1]. This age-related change in muscle quality, namely the proportion of contractile over non-contractile tissue, results in an impaired force-generating capacity and increased intramuscular fat [1], which eventually impact skeletal muscle function [2]. Additionally, muscle quality alterations are related to muscle thickness and the arrangement of muscles fibers, i.e., muscle architecture (MA) [3], which are strong determinants of muscle mass and muscle strength, respectively [4–6]. Therefore, the use of muscle quality and MA as biomarkers of skeletal muscle function, as well as for diagnostic and evaluation of exercise interventions, is important but relies on good reliability of the measurement procedure.

MA is quantified by fascicle length (FL), pennation angle (PA) and muscle thickness (MT), which are all affected by age [7–12]. For example, MT of the gastrocnemius medialis and vastus lateralis muscles is negatively correlated (*r* = − 0.4) with age [12], and both FL and PA of the gastrocnemius medialis muscle decline by ~ 10% by age 81 years [8]. Muscle status is often assessed by brightness-mode (B-mode) ultrasound (US) when priority is given to low-cost, rapid and non-invasive assessment [13–15]. Aggregate evidence suggests that US MA reliability estimates are highly variable and inconsistent in a variety of muscles and across studies [16–19]. One source of inconsistency is the use of incorrect types of intraclass correlation coefficients, which could inflate reliability or mask unreliability of MA parameters [16, 17, 19]. A second source of inconsistency is the use of reliability estimates of MA parameters that are not comparable between studies [16, 17, 19]. It is also often the case that due to a large number of images, two or more raters determine MA, however the reliability between raters analyzing the same image is scarcely reported [20]. Furthermore, reliability of US estimates of MA has only been scarcely examined in the older population. Therefore, the potential effects of aging on reliability, which results from the age-related alterations in muscle quality and architecture, remain unclear.

In fact, the age-related changes in muscle quality vary per individual, depend on co-existing pathologies and are reflected in sonographic changes, of which some of the most reoccurring ones are changes in echogenicity and loss of heterogeneity [21]. These changes concern, respectively, the ability of the tissue to reflect echo waves and the changes in the proportion of contractile and non-contractile tissue. Both these properties are quantifiable by ultrasound measures of muscle quality, in particular echo intensity (EI) [22]. Therefore, if both the MA reliability of lower limb muscles in aging and the effects of muscle quality on MA reliability remain unclear, the potential value of ultrasound for diagnostic and prognosis is also debatable. Additionally, since MA is a strong determinant of muscle strength [4–6], the conclusions on age-related loss of strength and low physical performance based on US measurements are less impactful, and so is the relationship between these metrics and muscle loss, or sarcopenia, which is associated with mobility-disability and mortality [23–26].

Although the use of US in clinical settings is promising [27], on the basis of the evidence presented, a clarification of the reliability of MA features for lower extremity muscles in older adults would increase the clinical value of MA and would allow clinicians to make more informed treatment decisions. Therefore, the purpose of this study is to provide a comprehensive analysis of relative and absolute intra-rater and inter-rater reliability of muscle architecture, namely FL, PA, and MT using panoramic two-dimensional (2D) US of three lower extremity muscles at rest and during contraction in healthy younger and older adults. The tibialis anterior, vastus lateralis and gastrocnemius medius muscles were targeted since their MA is affected by aging and because of the important role they play during balance control while standing [28, 29] and power production during walking [30, 31]

The second aim is to elucidate the effect of age on MA reliability and measures of muscle quality. Accordingly, the association between different MA parameters, will also be examined. This process would ultimately help reveal not only the structure of interdependence among MA parameters, but also facilitate the selection of those parameters that are reliable while being highly correlated to other MA parameters. We expect to find that reliability of MA parameters would vary by muscle. Furthermore, we expect significant positive correlations between FL and MT and FT and PA [32, 33]. As age affects muscles, connective tissue volume and intramuscular fat in the lower extremity muscles differently [34], which in turn affect muscle quality [35], we expect that reliability of MA would be lower in older compared with younger adults.

## Results

### Reliability of MA parameters

Table 1 shows the demographics and muscle property data.

**Table 1:** Demographic data and muscle properties in the two age groups

Variable	Young (*N* = 12)		Old (*N* = 12)	
Demographic data				
Age (years)	23.33 (3.75)		67.92 (2.11)	
Gender (male/female)	5/7		6/6	
Length (m)	1.76 (0.11)		1.70 (0.05)	
Mass (kg)	70.83 (11.46)		70.08 (13.47)	
Muscle architecture and echo intensity data				
Gastrocnemius medialis				
PA rest (°)	18.71 (3.12)		16.16 (2.91)	
PA contraction (°)	21.77 (3.45)		19.07 (5.17)	
FL rest (cm)	5.12 (0.75)		6.53 (2.91)	
FL contraction (cm)	4.51 (1.03)		5.34 (1.89)	
MT rest (cm)	1.63 (0.28)		1.61 (0.29)	
MT contraction (cm)	1.72 (0.42)		1.64 (0.30)	
Tibialis anterior				
PA rest (°)	9.82 (2.23)		11.81 (2.51)	
PA contraction (°)	13.10 (3.55)		14.88 (3.28)	
FL rest (cm)	6.26 (0.83)		6.51 (1.14)	
FL contraction (cm)	5.44 (0.90)		5.66 (0.91)	
MT rest (cm)	1.04 (0.21)		1.16 (0.17)	
MT contraction (cm)	1.17 (0.27)		1.28 (0.18)	
Vastus lateralis				
PA rest (°)	13.30 (1.92)		10.60 (2.79)	
PA contraction (°)	12.72 (3.30)		9.87 (2.52)	
FL rest (cm)	12.14 (2.12)		10.96 (3.81)	
FL contraction (cm)	11.18 (2.53)		11.90 (3.75)	
MT rest (cm)	2.71 (0.40)		1.98 (0.34)	
MT contraction (cm)	2.57 (0.34)		2.09 (0.23)	
MVF data (*N*)				
Gastrocnemius medialis	538.18 (259.73)		421.33 (153.97)	
Tibialis anterior	163.83 (66.59)		170.17 (54.98)	
Vastus lateralis	441.55 (152.80)		309.83 (102.03)	

Data are expressed as mean (SD)

*EI* echo intensity, *PA* pennation angle, *FL* fascicle length, *MT* muscle thickness, *MVF* maximum voluntary force

#### Intra-rater reliability

Table 2 shows the ICCs for each rater and ICC averaged over the three raters for PA, FL, and MT in the three muscles at rest and during contraction in the two age groups. All ICCs were significant. For the GM, average intra-rater ICCs were good to excellent (ICCs = 0.78–0.99) in the two age groups. Table 3 shows the corresponding absolute SEM and relative SEM values. Averaged intra-rater SEM values for the GM muscle were ≤ 1.84° for PA, ≤ 0.46 cm for FL and ≤ 0.05 cm for MT in both groups. For the TA, relative intra-rater reliability was moderate to excellent (ICCs = 0.70–0.98) in both age groups, and absolute SEM values were ≤ 1.24° for PA, ≤ 0.61 cm for FL and ≤ 0.04 cm for MT. For the VL, relative intra-rater reliability was moderate to excellent (ICCs = 0.59–0.98) in the two age groups. Corresponding absolute SEM values were ≤ 1.29° for PA in both groups. For FL, SEMs were ≤ 1.01 cm in the old group and ≤ 0.75 cm in the younger group. For MT, SEM values were ≤ 0.13 cm in the two age groups.

**Table 2:** Intra-rater intraclass correlations for muscle architecture in the three muscles and the two age groups

	Gastrocnemius medialis			Tibialis anterior			Vastus lateralis		
	PA	FL	MT	PA	FL	MT	PA	FL	MT
Young									
Rater 1									
Rest	0.77 (0.24–0.93)	0.70 (0.26–0.90)	0.98 (0.96–0.99)	0.79 (0.19–0.95)	0.56 (0.22–0.82)	0.98 (0.95–0.99)	0.86 (0.45–0.96)	0.93 (0.81–0.98)	0.96 (0.90–0.99)
Contraction	0.82 (0.30–0.95)	0.94 (0.73–0.98)	0.99 (0.98–1.00)	0.82 (0.40–0.95)	0.72 (0.43–0.90)	0.99 (0.97–1.00)	0.88 (0.42–0.97)	0.83 (0.50–0.95)	0.98 (0.94–0.99)
Rater 2									
Rest	0.71 (0.43–0.90)	0.80 (0.57–0.93)	0.97 (0.93–0.99)	0.72 (0.44–0.90)	0.74 (0.47–0.91)	0.96 (0.91–0.99)	0.62 (0.26–0.87)	0.82 (0.59–0.94)	0.93 (0.82–0.98)
Contraction	0.81 (0.57–0.94)	0.95 (0.87–0.98)	0.99 (0.97–1.00)	0.94 (0.84–0.98)	0.84 (0.64–0.94)	0.96 (0.89–0.99)	0.79 (0.54–0.93)	0.85 (0.67–0.95)	0.80 (0.57–0.93)
Rater 3									
Rest	0.91 (0.78–0.97)	0.78 (0.54–0.92)	0.99 (0.98–1.00)	0.83 (0.62–0.94)	0.79 (0.56–0.93)	0.99 (0.97–1.00)	0.70 (0.39–0.9)	0.81 (0.57–0.94)	0.98 (0.95–0.99)
Contraction	0.70 (0.39–0.90)	0.88 (0.71–0.96)	0.99 (0.98–1.00)	0.85 (0.66–0.95)	0.84 (0.64–0.95)	1.00 (0.99–1.00)	0.89 (0.74–0.96)	0.97 (0.92–0.99)	0.75 (0.49v0.91)
Mean									
Rest	*0.80*	*0.76*	*0.98*	*0.78*	*0.70*	*0.98*	*0.73*	*0.85*	*0.96*
Contraction	*0.78*	*0.92*	*0.99*	*0.87*	*0.80*	*0.98*	*0.85*	*0.88*	*0.84*
Old									
Rater 1									
Rest	0.83 (0.61–0.94)	0.98 (0.96–1.00)	0.98 (0.94–0.99)	0.82 (0.61–0.94)	0.85 (0.66–0.95)	0.96 (0.9–0.99)	0.86 (0.69–0.95)	0.84 (0.64–0.95)	0.97 (0.92–0.99)
Contraction	0.95 (0.88–0.98)	0.99 (0.98–1.00)	0.98 (0.96–1.00)	0.96 (0.91–0.99)	0.92 (0.79–0.97)	0.97 (0.92–0.99)	0.51 (0.17–0.8)	0.90 (0.77–0.97)	0.92 (0.81–0.97)
Rater 2									
Rest	0.69 (0.40–0.89)	0.88 (0.72–0.96)	0.95 (0.87–0.98)	0.89 (0.75–0.96)	0.80 (0.57–0.93)	0.94 (0.86–0.98)	0.57 (0.22–0.83)	0.81 (0.58–0.93)	0.94 (0.84–0.98)
Contraction	0.91 (0.78–0.97)	0.85 (0.65–0.95)	0.98 (0.95–0.99)	0.90 (0.75–0.97)	0.85 (0.66–0.95)	0.97 (0.93–0.99)	0.62 (0.30–0.86)	0.76 (0.50–0.92)	0.87 (0.70–0.96)
Rater 3									
Rest	0.82 (0.60–0.94)	0.98 (0.95–0.99)	0.99 (0.96–1.00)	0.90 (0.77–0.97)	0.54 (0.19–0.82)	0.93 (0.83–0.98)	0.63 (0.31–0.86)	0.81 (0.55–0.94)	0.98 (0.94–0.99)
Contraction	0.88 (0.72–0.96)	0.95 (0.88–0.98)	0.99 (0.98–1.00)	0.67 (0.37–0.88)	0.82 (0.61–0.94)	0.92 (0.81–0.98)	0.64 (0.32–0.87)	0.87 (0.71–0.96)	0.90 (0.77–0.97)
Mean									
Rest	*0.78*	*0.95*	*0.97*	*0.87*	*0.73*	*0.94*	*0.69*	*0.82*	*0.96*
Contraction	*0.91*	*0.93*	*0.98*	*0.84*	*0.86*	*0.95*	*0.59*	*0.84*	*0.90*

Data are expressed as intraclass correlations (ICCs) with 95% confidence intervals shown under each ICC value between brackets

*PA* pennation angle, *FL* fascicle length, *MT* muscle thickness

All ICCs: *P* < 0.05

**Table 3:** Intra-rater standard error of measurements for muscle architecture in the three muscles and the two age groups

	Gastrocnemius medialis			Tibialis anterior			Vatus lateralis		
	PA [°]	FL [cm]	MT [cm]	PA [°]	FL [cm]	MT [cm]	PA [°]	FL [cm]	MT [cm]
Young									
Rater 1									
Rest	1.55 (8.06)	0.38 (7.51)	0.03 (2.03)	1.17 (10.86)	0.57 (9.22)	0.03 (2.77)	0.79 (5.87)	0.57 (4.61)	0.08 (2.87)
Contraction	1.34 (6.15)	0.28 (6.07)	0.03 (1.96)	1.46 (10.49)	0.45 (8.23)	0.03 (2.67)	1.35 (10.45)	0.87 (7.76)	0.06 (2.15)
Rater 2									
Rest	1.53 (7.91)	0.30 (6.30)	0.05 (3.08)	1.08 (10.72)	0.40 (6.97)	0.04 (4.20)	1.32 (9.52)	0.58 (5.38)	0.11 (4.32)
Contraction	1.68 (7.79)	0.21 (4.94)	0.04 (2.45)	1.02 (7.25)	0.27 (5.31)	0.06 (4.84)	1.29 (9.55)	0.63 (6.43)	0.15 (6.26)
Rater 3									
Rest	1.17 (6.67)	0.44 (7.90)	0.02 (1.53)	0.92 (10.69)	0.37 (5.46)	0.02 (2.36)	1.15 (9.15)	1.11 (8.39)	0.05 (1.97)
Contraction	2.50 (11.45)	0.41 (8.95)	0.03 (1.90)	1.24 (10.96)	0.46 (7.83)	0.02 (1.64)	1.24 (10.55)	0.55 (4.43)	0.19 (7.17)
Mean									
Rest	1.42 (7.55)	0.37 (7.24)	0.03 (2.21)	1.06 (10.76)	0.45 (7.22)	0.03 (3.11)	1.09 (8.18)	0.75 (6.13)	0.08 (3.05)
Contraction	1.84 (8.46)	0.30 (6.65)	0.03 (2.10)	1.24 (9.57)	0.39 (7.12)	0.04 (3.05)	1.29 (10.18)	0.68 (6.21)	0.13 (5.19)
Old									
Rater 1									
Rest	1.32 (7.88)	0.30 (4.72)	0.04 (2.65)	1.01 (8.04)	0.34 (5.15)	0.04 (2.99)	1.02 (8.79)	0.75 (8.17)	0.07 (3.28)
Contraction	0.96 (5.20)	0.15 (2.74)	0.04 (2.32)	0.71 (4.43)	0.27 (4.60)	0.03 (2.62)	1.37 (13.43)	0.64 (5.86)	0.07 (3.39)
Rater 2									
Rest	1.65 (9.93)	0.55 (9.84)	0.07 (4.28)	0.92 (7.61)	0.48 (8.24)	0.04 (3.82)	1.30 (10.61)	0.59 (6.88)	0.08 (4.33)
Contraction	1.96 (9.37)	0.59 (12.70)	0.04 (2.67)	0.99 (6.64)	0.29 (5.67)	0.03 (2.57)	1.12 (9.28)	0.51 (5.69)	0.08 (3.89)
Rater 3									
Rest	1.39 (9.20)	0.53 (6.92)	0.04 (2.41)	0.80 (7.42)	1.00 (14.01)	0.05 (4.20)	1.40 (17.48)	1.70 (11.29)	0.05 (2.71)
Contraction	1.68 (9.40)	0.50 (8.56)	0.03 (1.65)	2.02 (14.75)	0.44 (7.32)	0.05 (3.75)	1.17 (15.92)	1.32 (8.37)	0.08 (3.56)
Mean									
Rest	1.45 (9.00)	0.46 (7.16)	0.05 (3.11)	0.91 (7.69)	0.61 (9.13)	0.04 (3.67)	1.24 (12.29)	1.01 (8.78)	0.07 (3.44)
Contraction	1.53 (7.99)	0.41 (8.00)	0.04 (2.21)	1.24 (8.61)	0.33 (5.86)	0.04 (2.98)	1.22 (12.88)	0.82 (6.64)	0.08 (3.61)

Data are expressed as standard error of measurement (SEM) with relative SEM values (SEM%) shown between brackets

*PA* pennation angle, *FL* fascicle length, *MT* muscle thickness

#### Inter-rater reliability

Table 4 shows the ICCs and SEMs for the inter-rater reliability for PA, FL, and MT in the three muscles at rest and during contraction in the two age groups. For the MA of the GM muscle, inter-rater reliability was good to excellent (ICCs = 0.76–0.99, all *P* < 0.05) in the two age groups. Absolute PA SEM values were below 1.83° in the younger group and ranged between 1.80° to 3.63°in the older group. FL SEMs were ≤ 0.55 cm in the younger group, and ranged between 1.03 and 1.84 cm in the older group. Absolute MT SEM values were ≤ 0.06 cm in the two age groups. Inter-rater reliability for TA MA was moderate to excellent (ICCs = 0.64–0.99, all *P* < 0.05) in the two age groups. SEM values for the PA, FL and MT of the TA were, respectively, ≤ 2.89°, ≤ 0.93 cm, and ≤ 0.05 cm. Inter-rater reliability for VL MA was moderate to excellent (ICCs = 0.67–0.98, all *P* < 0.05) in the younger group and poor to excellent in the older group (ICCs = 0.22–0.99). More specifically, in the older group, ICCs varied from 0.44 to 0.55 for PA, between 0.22 and 0.37 for FL, and between 0.94 and 0.99 for MT. However, the ICCs values for PA and FL in the older group were not significant. PA SEM values were ≤ 2.60° in the two age groups. FL SEM values were ≤ 4.05 cm in the older group and ≤ 2.04 cm in the younger group. SEM MT values were ≤ 0.17 cm in the younger group and ≤ 0.09 cm in the older group.

**Table 4:** Inter-rater intraclass correlation coefficients and standard errors of measurements for muscle architecture

ICCs	Gastrocnemius medialis			Tibialis anterior			Vastus lateralis		
	PA	FL	MT	PA	FL	MT	PA	FL	MT
Young									
Rest	0.86* (0.69–0.96)	0.76* (0.27–0.93)	0.98* (0.96–0.99)	0.85* (0.36–0.96)	0.64* (0.00–0.89)	0.98* (0.86–1.00)	0.72* (0.28–0.92)	0.81* (0.21–0.95)	0.98* (0.88–0.99)
Contraction	0.93* (0.80–0.98)	0.97* (0.91–0.99)	0.99* (0.97–1.00)	0.65* (0.13–0.89)	0.86* (0.37–0.96)	0.99* (0.97–1.00)	0.75* (0.37–0.92)	0.67* (0.13–0.90)	0.91* (0.72–0.97)
Old									
Rest	0.84* (0.57–0.95)	0.84* (0.53–0.95)	0.99* (0.97–1.00)	0.90* (0.65–0.97)	0.67* (0.08–0.90)	0.98* (0.92–1.00)	0.55 (− 0.11–0.86)	0.37 (− 0.13–0.77)	0.99* (0.96–1.00)
Contraction	0.77* (0.42–0.93)	0.88* (0.67–0.96)	0.99* (0.96–1.00)	0.85* (0.57–0.95)	0.86* (0.41–0.96)	0.97* (0.90–0.99)	0.44 (− 0.09–0.81)	0.22 (− 0.29–0.65)	0.94* (0.80–0.98)

SEMs	PA (°)	FL (cm)	MT (cm)	PA (°)	FL (cm)	MT (cm)	PA (°)	FL (cm)	MT (cm)
Young									
Rest	1.83 (9.79)	0.55 (10.73)	0.06 (3.82)	1.40 (14.25)	0.70 (11.22)	0.05 (4.70)	1.44 (10.84)	1.44 (11.88)	0.10 (3.87)
Contraction	1.52 (6.96)	0.31 (6.90)	0.06 (3.61)	2.89 (22.05)	0.54 (9.89)	0.04 (3.82)	2.37 (18.66)	2.04 (18.29)	0.17 (6.67)
Old									
Rest	1.80 (11.15)	1.84 (28.09)	0.06 (3.52)	1.29 (10.93)	0.93 (14.27)	0.04 (3.45)	2.60 (24.52)	3.95 (36.02)	0.07 (3.46)
Contraction	3.63 (19.06)	1.03 (19.37)	0.06 (3.70)	2.02 (13.57)	0.54 (9.58)	0.05 (4.09)	2.55 (25.83)	4.05 (34.04)	0.09 (4.52)

Intraclass correlations (ICCs) are expressed with 95% confidence intervals shown between brackets. Relative standard error of measurement (SEM%) are shown next to the Standard error of measurement (SEM)-value between brackets

*PA* pennation angle, *FL* fascicle length, *MT* muscle thickness

ICCS were *P* < 0.05 are highlighted with an *

### Echo intensity and correlations among MA parameters

**Fig. 1:**
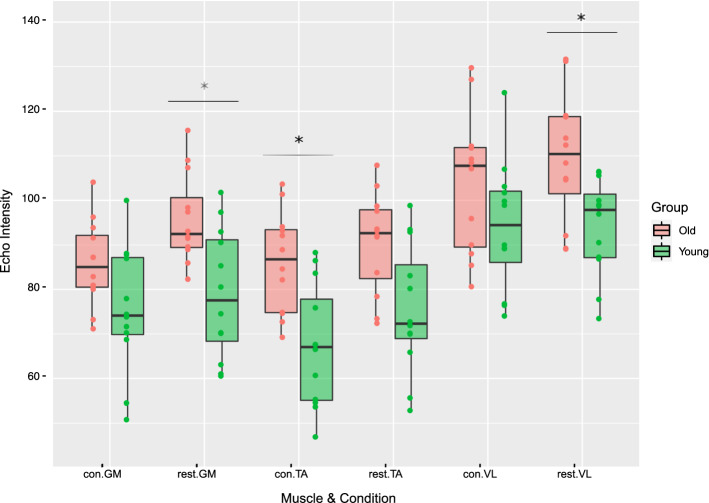
Echo intensity (EI) values in the three muscles and the two age groups. Significant (*P* < 0.05) EI differences between younger and older adults are highlighted with an *. *con* contraction, *GM* gastrocnemius medius, *TA* tibialis anterior, *VL* vastus lateralis

Figure 1 shows the echo intensity values at rest and during contraction in the three muscles and the two age groups. Mean and SD values of the echo intensity values are presented in Additional file 1: Table S1.

For the GM, EI in older adults was higher by 21.4% (Cohens’ *d* = 1.35) and 17.2% (Cohen’s *d* = 1.14) at rest and during contraction respectively, compared to younger adults. In older adults, TA EI was higher by 19.7% at rest (Cohens’ *d* = 1.14) and 28.0% (Cohens’ *d* = 1.47) during contraction, compared to younger adults. For the VL at rest, EI was 17.2% (Cohens’ *d* = 0.63) higher in older compared to younger adults, whereas during contraction it was 10.2% (Cohens’ *d* = 0.63) lower in younger adults. Large effects are reported in all muscles and conditions, except for the VL in contraction which showed a medium effect. Differences in EI between younger and older adults were only significant for GM in rest, TA in contraction and VL in rest (*P* < 0.05).

**Fig. 2:**
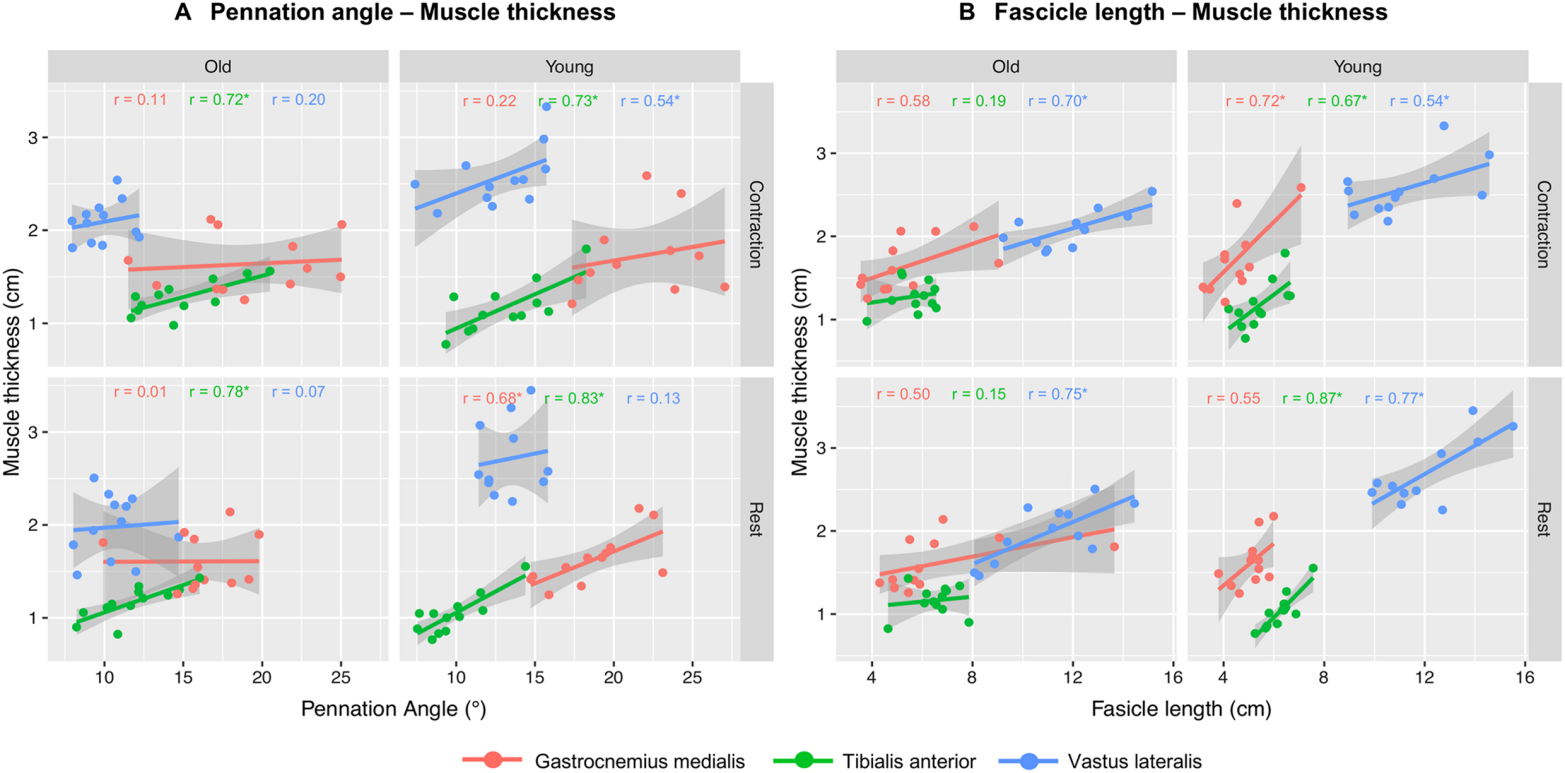
Correlations between muscle architecture (MA) parameters. **A** The correlations between pennation angle and muscle thickness in three muscles in rest and contraction and two age groups. **B** The correlations between fascicle length and muscle thickness in the three muscles at rest and contraction and in the two age groups. Significant correlations are highlighted with an *

Figure 2 shows the correlations between MA parameters at rest and during contraction in the three muscles and two age groups. In the younger group, GM, MT, and PA correlated moderately (*r* = 0.68, *P* < 0.05) at rest , but not during contraction (*r* = 0.22). In the older group, no correlations between MT and PA were found in the GM (*r*_rest_ = 0.01; *r*_con_ = 0.11). MT and PA correlated moderate to strong at rest and during contraction in TA in both groups (Young: *r*_rest_ = 0.83, *r*_con_ = 0.70; Old: *r*_rest_ = 0.78, *r*_con_ = 0.72, all *P* < 0.05). In VL, no correlation was found in the younger group at rest (*r* = 0.13), but a moderate correlation was found during contraction (*r* = 0.54, *P* < 0.05). MT and PA did not correlate in both conditions in older group (*r*_rest_ = 0.01, *r*_con_ = 0.11, respectively).

MT and FL in GM correlated similarly at rest in the younger (*r* = 0.55) and older group (*r* = 0.50), but different during contraction (young: *r* = 0.72, *P* < 0.05; old: *r* = 0.58). MT and FL in TA at rest and in contraction correlated in young (*r*_rest_ = 0.87, *r*_con_ = 0.67, both *P* < 0.05), but not in older adults (*r*_rest_ = 0.15, *r*_con_ = 0.19). MT and FL in VL at rest and during contraction correlated moderately to strongly in both age groups (young: *r*_rest_ = 0.77; *r*_con_ = 0.54; old: *r*_rest_ = 0.75; *r*_con_ = 0.70, all *P* < 0.05).

## Discussion

Age-related changes in MA parameters impact daily function. Reliable estimates of such measures are important for diagnostics and treatment of muscle-related disorders such as sarcopenia. A comprehensive reliability assessment of MA parameters in lower leg muscles has been lacking so far, especially in older adults. In the present study, we aimed to determine the relative and absolute intra-rater and inter-rater reliability of MA measured using 2D panoramic US in GM, TA, and VL at rest and during contraction. Furthermore, we wanted to elucidate the effects of age on both MA reliability and muscle quality, as well as on the interdependence of different MA parameters. As hypothesized, we found age- and muscle-specificity in the interdependence of MA parameters and in the reliability of MA parameters. Furthermore, as expected, muscle quality, expressed by EI, decreased with age, which could underlie the age-sensitivity of MA reliability estimates.

Overall, the values for FL, PA, and MT in the three muscles (Table 1) were within the limits of those reported previously [7, 8, 36]. Intra- and inter-rater reliability of the MA parameters was good to excellent in GM (ICCs: 0.76–0.99) and moderate to excellent in TA (ICCs: 0.64–0.99) in each age group (Tables 2, 4). In contrast, intra- and inter-rater reliability of MA parameters in VL was age-dependent, as it was moderate to excellent (ICCs: 0.67–0.98) in younger and poor to excellent (ICCs: 0.22–0.99) in older adults. These results are in line with the aggregate data reviewed previously [16–18]. However, when comparing our data with the data presented in these reviews, several points should be considered. First, the studies reviewed focused on test–retest, between scan or inter-operator ICCs. Second, different types or even incorrect reliability estimates were used and/or the type and model of the ICC were not reported. This may have inflated reliability or masked unreliability [16, 17, 19]. A study with a comparable methodology reported higher reliability (ICCs: 0.99–1.00) for MA parameters in GM [20]. These higher ICCs are probably the result of a longer probe being used which captured the entire length of the GM (100 mm [20] vs. 39 mm in the present study). Because panoramic US is sensitive to changes in position of the probe [37], our probe movement could have reduced the reliability. Furthermore, it is important to notice that in the current study ICCs were calculated based on absolute agreement, which could lower reliability compared to consistency measures. In the current study, SEM% values ranged from 2.1 to 12.9% for intra-rater reliability of PA, FL, and MT in the three muscles. For inter-rater reliability of the MA parameters, SEM% values ranged between 3.5–26.0% in the older group, and between 3.5–22.1% in the younger group.

### Comparison of reliability among MT, FL and PA

In all muscles, MT had the highest absolute and relative intra- and inter-rater reliability (ICCs > 0.87and SEM% < 7.2%) compared to PA (ICCs: 0.44–0.99; SEM%: 4.4–17.5%) and FL (ICCs: 0.22–0.99; SEM%: 2.7–14.0%). Additionally, MT confidence intervals were smaller than those of FL and PA, and did not overlap in most cases with the intervals of FL and PA, implying significant higher reliability for MT. As fascicles have variable lengths and arrangements within a muscle, the associated PA and FL may differ from fascicle to fascicle [4, 38], which may explain the lower reliability found for these two parameters.

### Comparison of reliability between rest and contraction

A previous study found improvements in image contrast and measurement accuracy for MA outcomes derived during contraction [39]. In the current study, we observed higher ICC estimates during muscle contraction compared with rest (~ 62%). However, when reliability of MA parameters was poor, as was the case for the FL and PA of the VL in older adults, reliability of MA parameters did not improve with contraction. Confidence intervals between rest and contraction were overlapping in most cases. Therefore, contraction does not seem to affect reliability of MA parameters.

### Comparison of reliability between the two age groups

Although ICC intervals are wide and overlap between both age groups, we observed that MA parameters were more variable in older compared to younger adults, in particular the PA and FL in VL, but not in GM and TA (Table 4). This sensitivity is reflected in the lower muscle quality in the older group (Fig. 2), possibly as a result of decreased muscle volume and increased fat infiltration [1, 35]. Furthermore, the larger volume of VL vs. GM and TA could have reduced probe stability during scanning [40], which influences image quality, since panoramic US is sensitive to probe re-positioning [37]. As older adults exhibit greater relative force fluctuations during isometric contractions [41, 42] and the large volume of VL lengthens scanning time, these force fluctuations could further compromise probe stability, image quality and ultimately the reliability of the VL MA. Our data also draw attention to using both ICC and SEM to assess reliability. While GM FL at rest had good reliability in both age groups (ICCs ≥ 0.76), SEM% were sensitive to age (young: 10.8%, old: 28.1%, Table 4), underlining the importance of assessing both absolute and relative reliability, especially when examining participants prone to muscle atrophy [16, 19].

### Muscle quality changes and correlations between MA parameters

As hypothesized, we observed that muscle quality, quantified using EI, was lower (*P* < 0.05) in older compared to younger adults [35]. The lower muscle quality in VL is consistent with the quadriceps’s lower quality in older adults reported in the literature [43, 44]. The lower muscle quality could have affected the ability to reliably identify the MA parameters, as it appears to be reflected in the larger range of the ICCs found in the older population. Therefore, the current results also highlight the relevance of a MA reliability analysis in an older population, including those having a specific disability that compromises muscle quality. Moreover, the MA data collected in three muscles and two age groups allowed us to examine the age- and muscle-specificity of the relationship among MT, PA, and FL. Such analyses could help us better understand the structural and functional mechanisms underlying senile sarcopenia and muscles’ responses to mechanical loading. This process also helps reveal the interdependence among MA parameters, and facilitates the selection of those parameters that have higher reliability while being highly correlated to other MA parameters. We observed, in agreement with our hypothesis and supported by prior data [32, 33], a muscle-specificity in the relationships between MT and PA, as there was such an association in GM (*r* = 0.68) and TA (*r* = 0.83) in younger adults, but not in the VL (*r* = 0.13) (Fig. 2). The MT–PA relationship was affected by age, as such association was only present in TA (*r* = 0.72, *P* < 0.05) in older adults, but not in GM (*r* = 0.01) and VL (*r* = 0.07). How age and specific muscles might affect the MT–FL relationship is important for understanding if muscle changes occur uniformly cross-sectionally (MT) and longitudinally (FL) [45]. We observed age- and muscle-specificity in the MT–FL relationship, so that such relationship occurred in both age groups in VL (Fig. 2. Younger: *r* = 0.75, *P* < 0.05, older: *r* = 0.70, *P* < 0.05), in neither group in GM (younger: *r* = 0.55; older: *r* = 0.50) and for the TA only in the younger adults (younger: *r* = 0.87, *P* < 0.05; older: *r* = 0.15). The data seem to suggest that old age affects the interdependence among measures of MA at rest. We note that muscle contraction did affect these associations between PA–MT and FL–MT for the younger (*r*_rest_ = 0.68; *r*_con_ = 0.22), but not for older adults. An explanation for this finding could be that we observed higher MVCs with more variation in the younger group for the GM and VL compared to the older group (Table 1). This indicates that there is also more variability in the absolute force level at which the muscles were contracted at 20–30% MVC. The selection of a MA measure that is highly correlated with other MA parameters while also being highly reliable may thus remain age- and muscle-dependent. However, on the basis of our analysis, we found that MT appears the simplest and most easily reproducible MA parameter (ICCs > 0.87, SEM% < 7.47%) in the older adults and it supports the use of MT in clinical settings, as done previously in the context of muscle strength research [46].

A greater sample size could have narrowed the 95% confidence intervals of the ICC in particular for the PA and FL of all muscles. Wider confidence intervals in the current study for PA and FL, and not for MT, have also been reported previously [47], when determining test–retest reliability of VL and GM muscles in 21 older adults. In the present study, FL was determined with a custom-made MATLAB tool, on 2D images, ignoring the curvature of the muscle which could have influenced reliability [48].

## Conclusions

The current study examined intra-rater and inter-rater reliability of muscle architecture in three lower limb muscles in healthy younger and healthy older individuals. Additionally, we clarified the effect of age on MA reliability and measures of muscle quality. In conclusion, we observed the presence of age- and muscle-specificity in the relationships between MT and PA and MT and FL at rest. Furthermore, we conclude that MA parameters can be reliably assessed with 2D panoramic US, but the level of reliability is likely influenced by muscle quality and varies with age, muscle, and MA measure. Among the MA parameters, MT appears to be the simplest and most easily reproducible MA parameter in older adults.

## Methods

### Participants

Healthy younger (*N* = 12, 5M, mean age: 23.3 SD: ± 3.8 years) and healthy older independently living volunteers (*N* = 12, 6M, age 67.9 ± 2.1 years) participated in the study. Exclusion criteria were: neurological disorders and orthopedic disabilities that limited mobility function, a hip or knee replacement in the last 3 years, inability to walk for 5 min without a walking aid, and a history of falls in the last year. The Local Ethical Committee approved the study protocol, which was executed in accordance with the declaration of Helsinki. Participants gave written informed consent prior to the start of the study.

### Muscle architecture measurements

In vivo MA of the GM, TA and VL muscles was examined by 2D B-mode US (Echoblaster, Telemed, Vilnius, Lithuania) with a 128-element linear-array probe (transducer field of view = 39 mm). The PanoView (Echowave II, Telemed, Lithuania) software was used to create panoramic images of the muscles. US settings were optimized to ensure the best contrast between muscle fascicles and background. The settings, including gain (70%), depth (50 mm for VL, 40 mm TA and GM) and frequency (8 MHz), were set prior to testing and held constant between participants and across trials. US scans were performed on the right leg.

To ensure the thickest part of the muscle was captured within the time limit for scanning, the proximal and distal ends of the most medial part of the muscle were identified, and the panoramic scan was made between ~ 20–80% of the total muscle length. The probe was moved along the longitudinal axis of the muscle oriented parallel to the muscle fascicles and perpendicular to the skin. Minimal pressure was applied with the probe on the skin to avoid muscle compression during scanning. Water-soluble transmission gel was applied to the skin to aid acoustic coupling. Directly after each trial the US scan was inspected and repeated if a movement artefact contaminated the image. For all three muscles, US scans were recorded both at rest and during 20–30% of maximal voluntary (MVC) contraction.

### Procedure

For all muscles, US scans were first made at rest. For the VL, US scans were made while the participant was seated on a custom-made chair [49] with the right leg strapped to a lever arm mounted on the chair with the knee and hip 90° flexed. For the analysis of the TA, participants sat on the seat of the dynamometer (KinCom AP125; Chattecx Inc., Chattanooga, TN., USA) with the back supported and the knee fully extended. The subject’s right foot was strapped to the foot plate attachment of the dynamometer. Two crossover upper-body belts and a thigh strap were used to minimize extraneous movements. The GM muscle was examined in a prone position on an examination table. Two straps were secured around the upper and lower leg to minimize extraneous movements. The subject’s right foot was secured to the foot plate attachment of the KinCom with the knee fully extended and the ankle at 90°. After a scan at rest, participants produced a weak and medium effort contraction of the muscle followed by an MVC with 30 s of rest between trials. To determine the MVC, participants contracted the quadriceps, plantar flexors and dorsiflexors, respectively, as rapidly and forcefully for 5 s. Afterwards the muscles were US-scanned during 20–30% of the participants’ MVC with the force target displayed on a monitor.

A total of 144 scans were collected (24 participants, 3 muscles, rest, contraction) by the same experimenter.

### Ultrasound data analysis

**Fig. 3:**
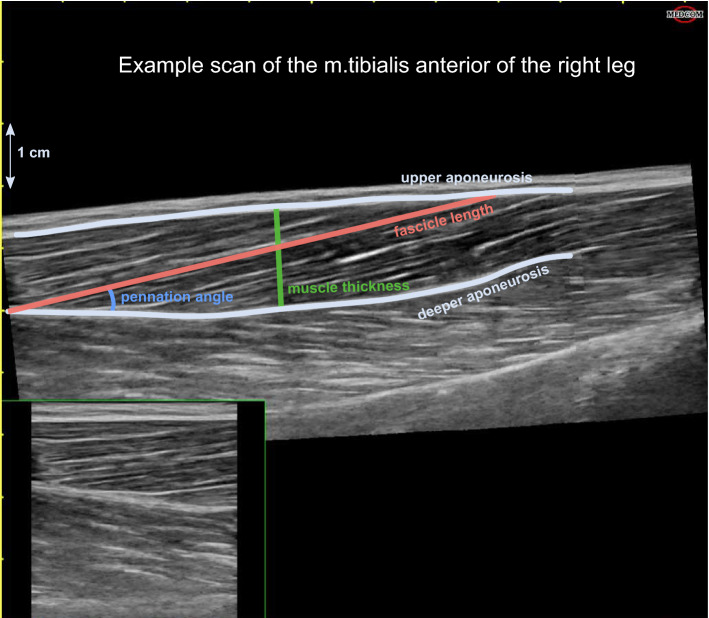
Representative ultrasound scan of the m. tibialis anterior at rest for a younger participant. The muscle architecture is color-annotated as follows, light blue/grey: upper and deeper aponeurosis; red: fascicle length; blue: pennation angle; green: muscle thickness

Using a custom-made graphical user interface in MATLAB (version r2019a; The MathWorks Inc., Natick, MA) PA, FL and MT were determined (Fig. 3). PA was defined as the angle between a clearly visible fascicle and the deep aponeurosis, FL as the length of the fascicular path between the deep and superficial aponeuroses [50] and MT as the perpendicular distance between the superficial and deep aponeuroses at the widest distance [46, 51]. FL and MT were converted from pixels to centimeters using the graduated scale on the original US scan.

To determine the absolute and relative reliability of the US scans, three raters with 6-month to 3-year experience on interpreting US scan images, analyzed the scans. All scans were presented three times [52] to each rater in random order. Due to a technical problem, data of one younger participant were missing for the VL at rest and for the GM during contraction. This led to a total of 630 ratings in the younger group and 648 ratings in the older group that were used for further statistical analyses.

**Fig. 4:**
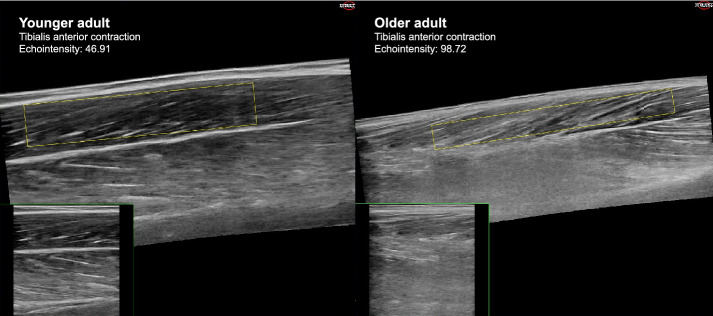
Example ultrasound scan of the m. tibialis anterior in contraction of a younger (left) and older adult (right). The yellow rectangles demonstrate the regions of interest selected for the echo intensity analysis. The mean pixel intensity of the regions of interested are displayed on the image

To assess whether the reliability of ultrasound is influenced by muscle quality, for each scan the EI was calculated. As non-contractile and contractile elements have different pixel intensities, where skeletal muscles appear black and intramuscular adipose and fibrous tissues appear white [53], EI can be used to examine muscle composition. For each ultrasound image, a region of muscle tissue of interest was selected with the exclusion of subcutaneous and fibrous tissue [22]. EI was than calculated as the mean pixel intensity of that region [22]. Figure 4 shows an ultrasound scan of the m. tibialis anterior during contraction of a younger (left) and older participant (right), with the regions of interest annotated in yellow and the mean EI values displayed at the top of the figure. EI values range between 0 and 255, with low values being indicative of good muscle quality [54].

### Statistical analysis

Statistical software (R version 3.6.1, R core team, 2019), including the IRR package (v0.84.1, Gamer, 2012) was used for all calculations. Intraclass correlation coefficients (ICC) with 95% confidence intervals were calculated as a measure of relative reliability, and the standard error of measurements (SEM) and its percentage (SEM%) were calculated as a measure of absolute reliability.

#### Reliability of MA parameters

Intra-rater reliability was determined by comparing the three ratings of the same image for each rater. Inter-rater reliability was determined by comparing the mean scores of the three ratings of each US scan among the three observers. For the intra-rater reliability, the ICC and SEM were based on a single-rating, absolute agreement, 2-way mixed-effects model with the following equation (for all reliability equations see [55]):

1 $$\frac{{\mathrm{MS}}_{\mathrm{R}}-{\mathrm{MS}}_{\mathrm{E}}}{{\mathrm{MS}}_{\mathrm{R}}+\left(k-1\right){\mathrm{MS}}_{\mathrm{E}}+\frac{k}{n}({\mathrm{MS}}_{\mathrm{C}}-{\mathrm{MS}}_{\mathrm{E}})},$$

with MS_R_ = mean square for rows, MS_E_ = mean square for error; MS_C_ = mean square for columns; *n* = number of subjects; *k* = number of measurements.

For the inter-rater reliability, the ICC and SEM were based on a mean-rating (*k* = 3), absolute agreement, 2-way random model with the following equation:

2 $$\frac{{\mathrm{MS}}_{\mathrm{R}}-{\mathrm{MS}}_{\mathrm{E}}}{{\mathrm{MS}}_{\mathrm{R}}+\frac{{\mathrm{MS}}_{\mathrm{C}}-{\mathrm{MS}}_{\mathrm{E}}}{n}}.$$

Reliability was classified as poor (ICC < 0.5), moderate (0.5 ≤ ICC ≤ 0.75), good (0.75 < ICC ≤ 0.9) or excellent (ICC > 0.9) [55]. To examine differences between ICCs of the different architectural parameters, younger and older adults, and rest and contraction, confidence intervals were compared [56]. The SEM for both models was calculated using the following equation [57]:

3 $$\mathrm{SEM}= \sqrt{{\mathrm{MS}}_{\mathrm{E}},}$$

where MS_E_ is the mean square error. A low SEM implies high reliability, though no generally accepted scales exist to interpret these values.

Additionally, SEM% was computed as follows [58]:

4 $$\mathrm{SEM}\%=\frac{\mathrm{SEM}}{\mathrm{mean}}*100.$$

#### Echo intensity and correlations among MA parameters

To examine whether measures of EI significantly differ between younger and older adults, independent *t*-tests were performed. To examine the relationship among the three MA parameters, MA parameters were first averaged across raters and trials and Pearson correlations coefficients were computed between the averaged MA values. Correlations were classified as weak (≤ 0.35), moderate (0.36–0.67), or strong (0.68–0.89) or very strong (≥ 0.90) [59]. For all analyses, statistical significance was set to *P* < 0.05.

## Supplementary Information

**Additional file 1: Table S1.** Echo intensity values in two age groups.

## Data Availability

The datasets used and/or analyzed during the current study are available from the corresponding author on reasonable request.

## Abbreviations

2D: Two-dimensional
B-mode: Brightness-mode
EI: Echo intensity
FL: Fascicle length
GM: Gastrocnemius medialis
ICC: Intraclass correlation coefficient
MA: Muscle architecture
MT: Muscle thickness
PA: Pennation angle
SEM: Standard error of measurement
SEM%: Relative standard error of measurement
TA: Tibialis anterior
US: Ultrasound
VL: Vastus lateralis

## References

[1] Doherty TJ (2003). Invited review: aging and sarcopenia. J Appl Physiol.

[2] Fragala MS, Kenny AM, Kuchel GA (2015). Muscle quality in aging: a multi-dimensional approach to muscle functioning with applications for treatment. Sport Med.

[3] Barbat-Artigas S, Rolland Y, Zamboni M, Aubertin-Leheudre M (2012). How to assess functional status: a new muscle quality index. J Nutr Health Aging.

[4] Lieber RL, Fridén J (2000). Functional and clinical significance of skeletal muscle architecture. Muscle Nerve.

[5] Rutherford OM, Jones DA (1992). Measurement of fibre pennation using ultrasound in the human quadriceps in vivo. Eur J Appl Physiol Occup Physiol..

[6] Selva Raj I, Bird SR, Shield AJ (2017). Ultrasound measurements of skeletal muscle architecture are associated with strength and functional capacity in older adults. Ultrasound Med Biol..

[7] Morse CI, Thom JM, Birch KM, Narici MV (2005). Changes in triceps surae muscle architecture with sarcopenia. Acta Physiol Scand.

[8] Narici MV, Maganaris CN, Reeves ND, Capodaglio P (2003). Effect of aging on human muscle architecture. J Appl Physiol..

[9] Stenroth L, Peltonen J, Cronin NJ, Sipilä S, Finni T (2012). Age-related differences in Achilles tendon properties and triceps surae muscle architecture in vivo. J Appl Physiol..

[10] Lexell J, Taylor CC, Sjöström M (1988). What is the cause of the ageing atrophy? Total number, size and proportion of different fiber types studied in whole vastus lateralis muscle from 15- to 83-year-old men. J Neurol Sci..

[11] Fukunaga T, Kubo K, Kawakami Y, Fukashiro S, Kanehisa H, Maganaris CN (2001). In vivo behaviour of human muscle tendon during walking. Proc R Soc Lond Ser B Biol Sci.

[12] Kubo K, Kanehisa H, Azuma K, Ishizu M, Kuno SY, Okada M (2003). Muscle architectural characteristics in women aged 20–79 years. Med Sci Sports Exerc..

[13] Narici M (1999). Human skeletal muscle architecture studied in vivo by non-invasive imaging techniques: functional significance and applications. J Electromyogr Kinesiol..

[14] Lieber RL, Ward SR (2011). Skeletal muscle design to meet functional demands. Philos Trans R Soc B Biol Sci..

[15] Narici MV, Binzoni T, Hiltbrand E, Fasel J, Terrier F, Cerretelli P (1996). In vivo human gastrocnemius architecture with changing joint angle at rest and during graded isometric contraction. J Physiol..

[16] English C, Fisher L, Thoirs K (2012). Reliability of real-time ultrasound for measuring skeletal muscle size in human limbs in vivo: a systematic review. Clin Rehabil.

[17] Nijholt W, Scafoglieri A, Jager-Wittenaar H, Hobbelen JSM, van der Schans CP (2017). The reliability and validity of ultrasound to quantify muscles in older adults: a systematic review. J Cachexia Sarcopenia Muscle..

[18] Kwah LK, Pinto RZ, Diong J, Herbert RD (2013). Reliability and validity of ultrasound measurements of muscle fascicle length and pennation in humans: a systematic review. J Appl Physiol..

[19] Hebert JJ, Koppenhaver SL, Parent EC, Fritz JM (2009). A systematic review of the reliability of rehabilitative ultrasound imaging for the quantitative assessment of the abdominal and lumbar trunk muscles. Spine (Phila Pa.

[20] McMahon JJ, Turner A, Comfort P (2016). Within- and between-session reliability of medial gastrocnemius architectural properties. Biol Sport..

[21] Walker FO, Cartwright MS, Wiesler ER, Caress J (2004). Ultrasound of nerve and muscle. Clin Neurophysiol..

[22] Stock MS, Thompson BJ (2021). Echo intensity as an indicator of skeletal muscle quality: applications, methodology, and future directions. Eur J Appl Physiol.

[23] Visser M, Goodpaster BH, Kritchevsky SB, Newman AB, Nevitt M, Rubin SM (2005). Muscle mass, muscle strength, and muscle fat infiltration as predictors of incident mobility limitations in well-functioning older persons. J Gerontol Ser A Biol Sci Med Sci.

[24] Janssen I, Heymsfield SB, Ross R (2002). Low relative skeletal muscle mass (Sarcopenia) in older persons is associated with functional impairment and physical disability. J Am Geriatr Soc.

[25] Visser M, Deeg DJH, Lips P, Harris TB, Bouter LM (2000). Skeletal muscle mass and muscle strength in relation to lower-extremity performance in older men and women. J Am Geriatr Soc.

[26] Cruz-Jentoft AJ, Pierre Baeyens J, Bauer JM, Boirie Y, Cederholm T, Landi F (2010). Sarcopenia: European consensus on definition and diagnosis Report of the European Working Group on Sarcopenia in Older People. Age Ageing..

[27] Tosato M, Marzetti E, Cesari M, Savera G, Miller RR, Bernabei R (2017). Measurement of muscle mass in sarcopenia: from imaging to biochemical markers. Aging Clin Exp Res.

[28] Afschrift M, de Groote F, Jonkers I (2021). Similar sensorimotor transformations control balance during standing and walking. PLoS Comput Biol..

[29] Papegaaij S, Baudry S, Négyesi J, Taube W, Hortobágyi T (2016). Intracortical inhibition in the soleus muscle is reduced during the control of upright standing in both young and old adults. Eur J Appl Physiol..

[30] Waanders JB, Murgia A, Hortobágyi T, DeVita P, Franz JR (2020). How age and surface inclination affect joint moment strategies to accelerate and decelerate individual leg joints during walking. J Biomech..

[31] DeVita P, Hortobagyi T (2000). Age causes a redistribution of joint torques and powers during gait. J Appl Physiol..

[32] Abe T, Brechue WF, Fujita S, Brown JB (1998). Gender differences in FFM accumulation and architectural characteristics of muscle. Med Sci Sports Exerc..

[33] Kawakami Y, Abe T, Kanehisa H, Fukunaga T (2006). Human skeletal muscle size and architecture: variability and interdependence. Am J Hum Biol.

[34] Degens H, Korhonen MT (2012). Factors contributing to the variability in muscle ageing. Maturitas..

[35] Strobel K, Hodler J, Meyer DC, Pfirrmann CWA, Pirkl C, Zanetti M (2005). Fatty atrophy of supraspinatus and infraspinatus muscles: accuracy of US. Radiology..

[36] de Boer MD, Seynnes OR, di Prampero PE, Pišot R, Mekjavić IB, Biolo G (2008). Effect of 5 weeks horizontal bed rest on human muscle thickness and architecture of weight bearing and non-weight bearing muscles. Eur J Appl Physiol..

[37] Weng L, Tirumalai AP, Lowery CM, Nock LF, Gustafson DE, Von Behren PL (1997). US extended-field-of-view imaging technology. Radiology..

[38] Gans C, de Vree F (1987). Functional bases of fiber length and angulation in muscle. J Morphol..

[39] Mairet S, Maïsetti O, Portero P (2006). Homogeneity and reproducibility of in vivo fascicle length and pennation determined by ultrasonography in human vastus lateralis muscle. Sci Sport..

[40] Giannakou E, Aggeloussis N, Arampatzis A (2011). Reproducibility of gastrocnemius medialis muscle architecture during treadmill running. J Electromyogr Kinesiol..

[41] Tracy BL, Enoka RM (2002). Older adults are less steady during submaximal isometric contractions with the knee extensor muscles. J Appl Physiol..

[42] Bazzucchi I, Felici F, Macaluso A, De Vito G (2004). Differences between young and older women in maximal force, force fluctuations, and surface emg during isometric knee extension and elbow flexion. Muscle Nerve.

[43] Abe T, Sakamaki M, Yasuda T, Bemben MG, Kondo M, Kawakami Y (2011). Age-related, site-specific muscle loss in 1507 Japanese men and women aged 20 to 95 years. J Sport Sci Med.

[44] Maden-Wilkinson TM, Degens H, Jones DA, McPhee JS (2013). Comparison of MRI and DXA to measure muscle size and age-related atrophy in thigh muscles. J Musculoskelet Neuronal Interact.

[45] Narici MV, Maganaris CN (2007). Plasticity of the muscle-tendon complex with disuse and aging. Rev..

[46] Strasser EM, Draskovits T, Praschak M, Quittan M, Graf A (2013). Association between ultrasound measurements of muscle thickness, pennation angle, echogenicity and skeletal muscle strength in the elderly. Age (Omaha)..

[47] Raj IS, Bird SR, Shield AJ (2012). Reliability of ultrasonographic measurement of the architecture of the vastus lateralis and gastrocnemius medialis muscles in older adults. Clin Physiol Funct Imaging..

[48] Noorkoiv M, Stavnsbo A, Aagaard P, Blazevich AJ (2010). In vivo assessment of muscle fascicle length by extended field-of-view ultrasonography. J Appl Physiol..

[49] dos Santos PCR, Hortobágyi T, Zijdewind I, Bucken Gobbi LT, Barbieri FA, Lamoth C (2019). Minimal effects of age and prolonged physical and mental exercise on healthy adults’ gait. Gait Posture..

[50] Baroni BM, Geremia JM, Rodrigues R, De Azevedo Franke R, Karamanidis K, Vaz MA (2013). Muscle architecture adaptations to knee extensor eccentric training: *Rectus femoris* vs. vastus lateralis. Muscle Nerve.

[51] Palmer TB, Akehi K, Thiele RM, Smith DB, Thompson BJ (2015). Reliability of panoramic ultrasound imaging in simultaneously examining muscle size and quality of the hamstring muscles in young, healthy males and females. Ultrasound Med Biol..

[52] Koppenhaver SL, Parent EC, Teyhen DS, Hebert JJ, Fritz JM (2009). The effect of averaging multiple trials on measurement error during ultrasound imaging of transversus abdominis and lumbar multifidus muscles in individuals with low back pain. J Orthop Sports Phys Ther..

[53] Pillen S, Tak RO, Zwarts MJ, Lammens MMY, Verrijp KN, Arts IMP (2009). Skeletal muscle ultrasound: correlation between fibrous tissue and echo intensity. Ultrasound Med Biol..

[54] Young HJ, Southern WM, Mccully KK (2016). Comparisons of ultrasound-estimated intramuscular fat with fitness and health indicators. Muscle Nerve..

[55] Koo TK, Li MY (2016). A guideline of selecting and reporting intraclass correlation coefficients for reliability research. J Chiropr Med.

[56] Kowalik D, Choi YH, Zou GY (2011). Confidence interval estimation for a difference between two dependent intraclass correlation coefficients with variable class sizes. J Stat Theory Pract.

[57] de Vet HCW, Terwee CB, Knol DL, Bouter LM (2006). When to use agreement versus reliability measures. J Clin Epidemiol..

[58] Lexell JE, Downham DY (2005). How to assess the reliability of measurements in rehabilitation. Am J Phys Med Rehabil..

[59] Taylor R (1990). Interpretation of the correlation coefficient: a basic review. J Diagn Med Sonogr.

