# Supplementary material for: Age- and muscle-specific reliability of muscle architecture measurements assessed by two-dimensional panoramic ultrasound
Source: Biomed Eng Online. 2022 Feb 13;21:15. doi: 10.1186/s12938-021-00967-4 (PMC8842860; doi:10.1186/s12938-021-00967-4)
Supplement: Supplementary file 1 — Additional file 1: Table S1. Echo intensity values in two age groups. [file 12938_2021_967_MOESM1_ESM.docx]

**Additional data**

**Table S1.** Echo Intensity values in two age groups

| **Echo Intensity** | **Young**  **(N=12)** | | **Old**  **(N=12)** | |
| --- | --- | --- | --- | --- |
| *Gastrocnemius medialis* |  |  |  |  |
| rest | 79.0 | (14.5) | 95.9 | (10.1) |
| contraction | 73.2 | (12.4) | 85.8 | (9.59) |
| *Tibialis anterior* |  |  |  |  |
| rest | 75.6 | (14.5) | 90.5 | (11.3) |
| contraction | 67.2 | (13.8) | 86.0 | (11.4) |
| *Vastus lateralis* |  |  |  |  |
| rest | 93.5 | (11.4) | 109.6 | (14.6) |
| contraction | 94.2 | (14.6) | 103.8 | (15.9) |

Data are expressed as mean (SD)
